# A Residual-Inception U-Net (RIU-Net) Approach and Comparisons with U-Shaped CNN and Transformer Models for Building Segmentation from High-Resolution Satellite Images

**DOI:** 10.3390/s22197624

**Published:** 2022-10-08

**Authors:** Batuhan Sariturk, Dursun Zafer Seker

**Affiliations:** Department of Geomatics Engineering, Faculty of Civil Engineering, Istanbul Technical University, Istanbul 34469, Turkey

**Keywords:** building segmentation, CNN, Transformer, Inception, residual connections, satellite images

## Abstract

Building segmentation is crucial for applications extending from map production to urban planning. Nowadays, it is still a challenge due to CNNs’ inability to model global context and Transformers’ high memory need. In this study, 10 CNN and Transformer models were generated, and comparisons were realized. Alongside our proposed Residual-Inception U-Net (RIU-Net), U-Net, Residual U-Net, and Attention Residual U-Net, four CNN architectures (Inception, Inception-ResNet, Xception, and MobileNet) were implemented as encoders to U-Net-based models. Lastly, two Transformer-based approaches (Trans U-Net and Swin U-Net) were also used. Massachusetts Buildings Dataset and Inria Aerial Image Labeling Dataset were used for training and evaluation. On Inria dataset, RIU-Net achieved the highest IoU score, F1 score, and test accuracy, with 0.6736, 0.7868, and 92.23%, respectively. On Massachusetts Small dataset, Attention Residual U-Net achieved the highest IoU and F1 scores, with 0.6218 and 0.7606, and Trans U-Net reached the highest test accuracy, with 94.26%. On Massachusetts Large dataset, Residual U-Net accomplished the highest IoU and F1 scores, with 0.6165 and 0.7565, and Attention Residual U-Net attained the highest test accuracy, with 93.81%. The results showed that RIU-Net was significantly successful on Inria dataset. On Massachusetts datasets, Residual U-Net, Attention Residual U-Net, and Trans U-Net provided successful results.

## 1. Introduction

With the developments in satellite and remote sensing technologies, building segmentation and generating building maps have become important research topics in recent years [1]. Due to these developments, high-resolution satellite images have become even more accessible and convenient data sources [2]. As an important feature in the urban environment, the mapping of buildings has significant importance for different applications such as urban mapping, population estimation, land cover/land use analysis, cadastral and topographic map production, change detection, and disaster management [3,4,5,6]. Nonetheless, obtaining reliable and accurate building maps from high-resolution satellite images is still challenging due to various reasons such as complex backgrounds [7,8], similarities between the background and the buildings [9], noise in data [4], heterogeneity in data structures [7,8], diversity in roof types [10], and characteristics (size, shape, color, etc.) of buildings [11], and other topological difficulties. If the problems encountered according to the methods used are briefly mentioned, the conventional methods that were used in the early studies generally use manually extracted features. In these models, the extraction process requires prior knowledge, which leads to a poor model generalization ability and is costly and time-consuming [7]. Deep learning methods that later replaced these methods in the following years have their own concerns. For example, convolutional neural networks (CNN) perform regional divisions, use expensive fully connected layers, and they lose accuracy and details due to stacked convolution layers [12]. Fully convolutional networks (FCN) use fixed-size convolutions that result in a local receptive field; thus, they lack the ability to model in the global context [13]. Transformers, which have been used frequently in recent years, are computationally inefficient and need large amounts of memory and big datasets [6].

Over the past few years, many researchers have tried various methods to achieve automatic building segmentation [1]. Among these methods, vision-based methods used for various studies ranging from deformation detection [14] and fraud detection to image segmentation have come to the fore. In conventional vision methods, manually extracted features including geometrical, spatial, and spectral information, and low-level features such as shape, color, edge, texture, and shadow are used [15]. These methods generally utilize these manually extracted features and apply classifiers or traditional machine learning techniques (e.g., Random Forests, Boosting, and Support Vector Machines) to achieve building segmentation [13]. However, extracting these features requires prior knowledge and is labor-intensive [16]. Although these methods have realized some progress, they have shortcomings, such as low accuracy, low generalization ability, and complex processing [3]. Due to the complex structures of buildings and similarities with other ground object categories (e.g., roads and cement floors), the predictions highly depend on the adjustment and feature design. With big data and the development of new algorithms and powerful hardware, deep learning methods have become common and gained a lot of traction in the remote sensing and computer vision communities.

Over the years, deep learning methods have gradually replaced conventional methods in image segmentation studies and achieved breakthroughs [13]. With the rapid development in computing powers and the increasing availability of data sources, deep learning methods, particularly CNNs, have started to be widely used as they surpass the traditional methods in terms of efficiency and accuracy [7]. Different from the conventional methods, CNNs have the capacity to extract features directly from the inputs and make predictions using sequential convolutions with fully connected layers [4]. CNNs can therefore be considered as one-step techniques that combine feature extraction and image classification into a single model. Hence, they also possess a good generalization ability [4]. Many researchers have used CNN architectures such as AlexNet [17], LeNet [18], VGGNet [19], ResNet [20], and GoogleNet [21]. However, these CNNs perform region divisions and use computationally expensive fully connected layers [3]. In earlier studies, CNN models that were patch-based had achieved exceptional success in building segmentation; however, they were unable to guarantee the integrity and the spatial continuity of building features due to their reliance on small patches around target features to make predictions and ignoring the relationships between them [4]. Although the advances in CNNs have promoted research in the area, there still exist some challenges. For example, differences in building size, shape, and colors, geometric complexity, and high in-class and low inter-class variance make it hard to extract and segment buildings from high-resolution satellite images. Therefore, traditional CNNs are not suitable for segmentation studies from satellite images [4].

To improve the performance of CNNs, Long et al. [22] presented the FCN, which have convolution layers instead of fully connected layers, which improved training and prediction accuracy to a great extent. FCNs can output feature maps at the size of the input images using upsampling, and achieve pixel-based segmentation through an encoder–decoder structure. Small-sized feature maps are generated in the encoder path via downsampling to gather semantic information. In the decoder path, final segmentations are obtained by decoding the feature maps. However, the segmentation is not accurate enough, due to the FCN having only one upsampling layer, and much information is lost in the decoder [16].

Consequently, FCNs have become effective methods for image segmentation studies, and various variants have emerged, such as U-Net [23] and SegNet [24], to further improve its performance and efficiency. U-Net improves image segmentation accuracy by concatenating the semantic information through skip connections, implementing upsampling step by step, and integrating same scale downsampling in each step [23]. SegNet has a convolutional encoder with a pooling layer and a symmetrical decoder with transposed convolutions, which reduces the number of training parameters [24].

These CNN-based approaches have achieved successful results, but they also have some bottlenecks. For example, using a fixed-size convolution results in a local receptive field. CNNs are designed to successfully extract local context, and are inherently low in capacity to extract global context [13]. To capture the global context, the most widely used approach is to implement the attention mechanism into the networks [25]. Several studies introduced the attention mechanism to improve feature representation; thereby, they can differentiate buildings from complex backgrounds [26,27]. Deng et al. [28] proposed a grid-based attention mechanism, Guo et al. [29] developed a parallel attention module, Pan et al. [30] tried to combine channel and spatial attention to improve the accuracy for building segmentation, and Cai and Chen [31] implemented a multi-path hybrid attention to improve the performance of segmenting small buildings. However, all these attention-based approaches rely on convolution operations to a great extent, and still have some limitation in global modeling [13]. Furthermore, numerous studies have been published to enhance the building segmentation performance of the models by concentrating on the network architectural design. Along with the attention mechanisms, approaches such as deep and shallow networks [32], multiple receptive fields, and residual connections [33] have been widely used.

CNNs have shown high performance on many image segmentation tasks. Despite their success, their low efficiency in capturing global context information still poses a challenge for researchers. Some studies have used the self-attention mechanism to overcome these issues; however, computational complexity grows with the spatial size, and hence, they may be suitable only for low-resolution images [34].

The Transformer approach was first proposed to be used in natural language processing (NLP), but has recently attracted interest in the computer vision community [6]. The first self-attention-based Vision Transformer (ViT) was proposed in 2020 [35]. It obtained competitive results on the ImageNet dataset and became an engaging approach in computer vision tasks. Different from CNNs, ViT takes 2D images, translates them into 1D sequences, and uses self-attention for feature characterization. Considering the advantage of this design, ViT shows superiority over CNNs in global feature extraction [13]. ViT-based approaches have made a tremendous job for image segmentation studies [36,37,38]. Many researchers in the computer vision and remote sensing field have implemented these methods for segmentation tasks, such as urban monitoring [39,40], land cover/land use analysis [41,42], change detection [43,44], and building segmentation [12,45]. Chen et al. [6] introduced a Sparse Token Transformer to learn the global dependency of Transformer tokens, Yuan and Xu [16] implemented Swin Transformer as an encoder and designed a multi-scale adaptive decoder, and Wang et al. [13] proposed a ViT-based dual-path structure named BuildFormer.

CNNs can only pay attention to small range of neighborhood features, and they are insufficient in caring global features. Transformers can compensate for these shortcomings of CNNs by using the attention mechanism approach. In ViT-based methods, in contrast to CNNs, global information is extracted; however, spatially detailed context is ignored. In addition, when working with the large-sized, high-resolution images, their vector operations use all the pixels, and these operations are generally computationally inefficient and need a large amount of memory [6]. To deal with these issues, the Swin Transformer approach [46] utilizes shifted window-based multi-head self-attention, but the complexity still increases with the window size.

While Transformers perform well in global context modeling, they have exerted some limitations in fine-grained detail capturing [34]. To use the advantages of both sides, efforts have been paid to combine CNNs and Transformers, and Trans U-Net [47] was proposed. This approach initially utilizes CNN to extract low-level features, and then uses Transformer to model global interactions.

In this study, a total of 10 CNN and Transformer models were generated, and building segmentation from high-resolution satellite images was carried out. Alongside our proposed approach Residual-Inception U-Net (RIU-Net), U-Net, Residual U-Net, and Attention Residual U-Net models, four state-of-the-art CNN architectures (Inception, Inception-ResNet, Xception, and MobileNet) were implemented as encoders to U-Net-based CNN models. Furthermore, two Transformer-based approaches (Trans U-Net and Swin U-Net) were also used within the study, and comparisons were realized. RIU-Net is designed to cope with Transformer architectures that have achieved successful results in recent years, and deal with the issues that CNNs and Transformers are facing, by modernizing frequently used CNN approaches towards the ViT design approach. It is aimed to obtain an up-to-date CNN approach by using modern methods such as Layer Normalization and GELU activation function inspired by the study from Liu et al. [48], together with modern and successful approaches such as Inception, residual connection, and asymmetric convolutions. There are many publicly available datasets that can be used for building segmentation, e.g., Massachusetts Buildings Dataset [49], Inria Aerial Image Labeling Dataset [50], and WHU Building Dataset [51]. The Massachusetts Buildings Dataset and the Inria Aerial Image Labeling Dataset were used in this study to train and test the created models. Performance evaluations for the models were completed using evaluation metrics F1 and Intersection over Union (IoU) scores, and the acquired results were discussed. The study aims to make comparisons between several CNN and Transformer-based models, and to see the position of our proposed RIU-Net model among the others, in terms of forming a solution to the above-mentioned problems, for building segmentation from high-resolution satellite images. The main contributions of this study can be summarized as below:

- A novel CNN architecture approach, modernized towards the ViT design, named RIU-Net, has been proposed.
- To cope with Transformers and the issues that CNNs are dealing with, our proposed architecture uses approaches and methods such as a u-shaped design, Inception, residual connections, skip connections, asymmetric convolutions, GELU, and Layer Normalization together.
- Comparisons between our proposed approach and several state-of-the-art CNN and Transformer approaches have been realized using two different publicly available building segmentation datasets that contain high-resolution satellite images.
- It has been observed that the RIU-Net approach is significantly successful when sufficient data are provided, especially on datasets containing complex buildings with different characteristics.

## 2. Materials and Methods

### 2.1. Datasets

Within the study, two publicly available building segmentation datasets were used. These datasets selected are: Inria Aerial Image Labeling Dataset and Massachusetts Buildings Dataset.

#### 2.1.1. Inria Aerial Image Labeling Dataset

Inria Aerial Image Labeling Dataset [50] features RGB, orthorectified images with a 30 cm spatial resolution. It covers a total area of 810 km${}^{2}$ and is split into two sets, training and testing. There is a corresponding labeled mask for each image in the training set, containing two classes, which are building and non-building. Since only the masks of the images in the training set are available publicly, these data were used in this study.

The Inria dataset includes images from different cities, extending from rural to urban areas, with various building characteristics. A total of 180 images and corresponding masks from five cities are included in the training set (36 images and masks from each city). The cities are; Western Tyrol (Austria), Vienna (Austria), Kitsap County (WA, USA), Chicago (IL, USA), and Austin (TX, USA) [50]. Each image and mask are 5000 × 5000 pixels and cover an area of 1500 m × 1500 m. The use of these data provides an increase in the generalization capabilities of the models, since they contain images with different building characteristics from various different regions.

#### 2.1.2. Massachusetts Buildings Dataset

Massachusetts Buildings Datasets were introduced by Volodymyr Mnih [49] in 2013, as a part of his Ph.D. thesis. This dataset consists of 151 RGB images and their labeled masks of Boston, MA, USA. Every image and mask in the dataset is 1500 × 1500 pixels in size, has a spatial resolution of 1 m, and spans a 2.25 km${}^{2}$ area. This dataset covers mostly suburban and urban areas, which include buildings with similar characteristics and different sizes. The building labels for masks were obtained by digitizing buildings from the OpenStreetMap project [49].

#### 2.1.3. Preparation of the Data

**Figure 1:**
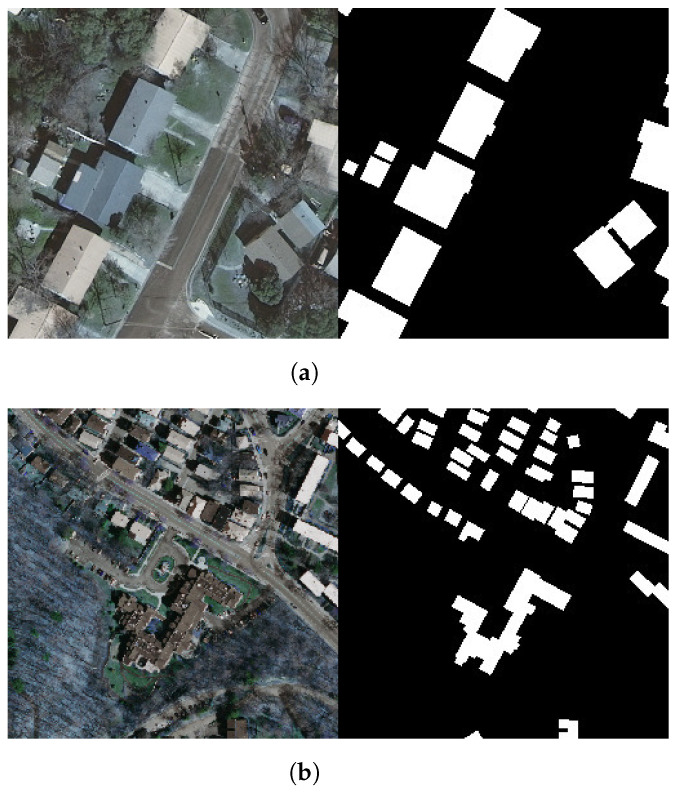
Sample 256 × 256 pixel image and mask: (**a**) Inria dataset, (**b**) Massachusetts dataset.

To prepare the datasets to be used in the study, the images and corresponding masks from both datasets were initially cropped into 256 × 256 pixel-sized patches, to reduce the computational cost. After the cropping process, images with no buildings or few buildings were removed from the datasets. From the Inria dataset, 7500 images and corresponding masks were obtained. Out of these images, 70% (5250) were selected as the training set, 15% (1125) as the validation set, and 15% as the test set. From the Massachusetts dataset, after the cropping and eliminations, only 488 images and their masks were obtained. Since the quantity of images is insufficient for this study, “brightness adjustment” and “rotation” data augmentation techniques have been used on the images. The brightness of the images was raised by a gamma rate of 0.5, and they were rotated 90° clockwise. After these operations, two datasets were obtained, named as Massachusetts Small and Massachusetts Large. Massachusetts Small dataset contains a total of 1500 images and masks, whereas Massachusetts Large dataset contains 3041 images and masks. Similar to the Inria dataset, 70% of the data for both datasets were selected as the training set, 15% as the validation set, and 15% as the test set. Consequently, three datasets were prepared using the Inria and the Massachusetts datasets (Figure 1).

### 2.2. Methodology

A total of 10 CNN and Transformer models were generated and used for building segmentation from high-resolution satellite images. Alongside our proposed approach Residual-Inception U-Net (RIU-Net), U-Net, Residual U-Net, and Attention Residual U-Net models, four state-of-the-art CNN architectures, which are Inception, Inception-ResNet, Xception, and MobileNet, were implemented as encoders to the U-Net-based CNN models. Moreover, two Transformer-based approaches, Trans U-Net and Swin U-Net, completed the model tree used in this study.

#### 2.2.1. U-Net

U-Net is an FCN-based architecture proposed by Ronneberger et al. [23]. It has a symmetrical, u-shaped design that includes an encoder, a decoder, and bottleneck paths. The encoder extracts relevant features and these extracted features propagate to the decoder using skip connections. Afterward, the decoder reconstructs the images into desirable dimensions using these feature maps. The bottleneck path is placed between the encoder path and the decoder path, and contains two 3 × 3 convolution layers. There are four convolution blocks in the encoder path, and each one includes two 3 × 3 convolution layers with Rectified Linear Unit (ReLU) activation, and a 2 × 2 max pooling layer. The decoder path also has of four blocks, with each block having one 2 × 2 transposed convolution layer, a concatenation layer to connect the extracted feature maps, and two 3 × 3 convolution layers [23].

#### 2.2.2. Residual U-Net

The Residual connection was introduced by He et al. [20] via ResNet architecture to cope with the problems of deep CNNs. When a model has more layers added on, it experiences the “vanishing gradient problem”. With the help of the residual connections, spatial information can be passed layers down directly, to cope with this problem.

**Figure 2:**
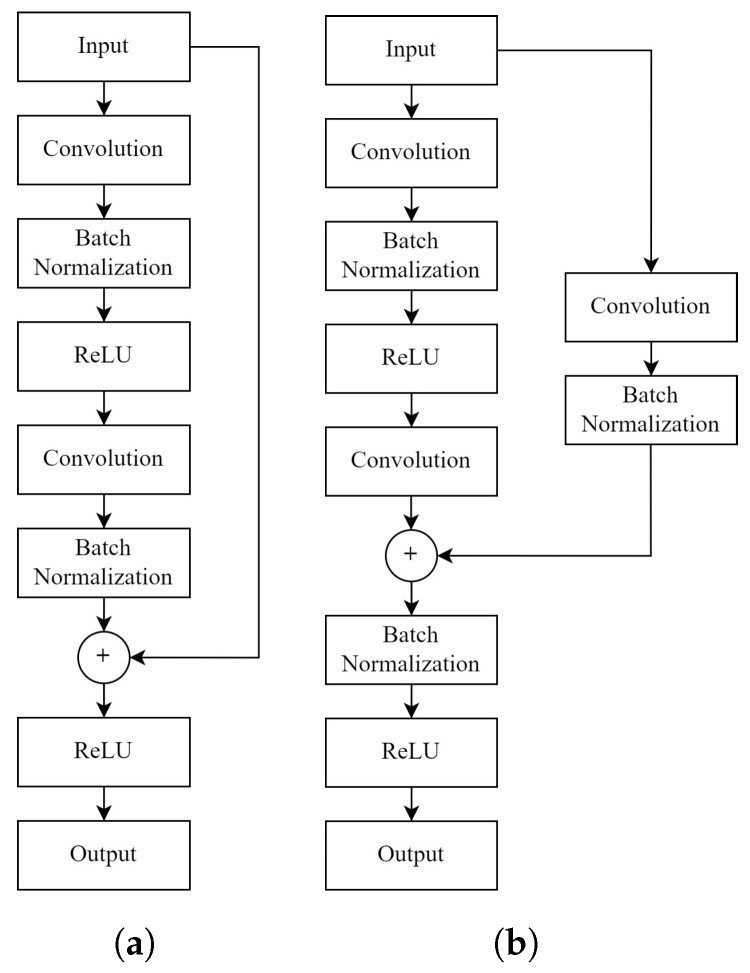
(**a**) Residual connection design from the ResNet. (**b**) Residual connection design implemented in the study.

In this study, a Residual U-Net model was generated. The model combines residual connections with the U-Net architecture by replacing conventional convolutional blocks in the U-Net with residual convolutional blocks. In this model, another variant was used instead of the residual connection design used in ResNet. Several different designs have been tried and the design proposed by He et al. [52], shown in Figure 2, was chosen, and it was modified to replace the conventional convolutional block in U-Net.

#### 2.2.3. Attention Residual U-Net

**Figure 3:**
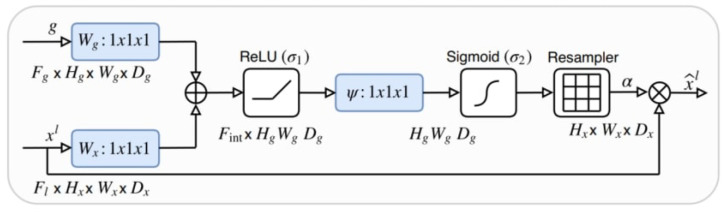
Attention mechanism implemented in the study [26].

In addition to the Residual U-Net model, an Attention Residual U-Net model was also utilized within the study. This approach combines the Residual U-Net model with the attention mechanism to achieve performance improvements. Through this implementation, the model could focus more on relevant features and produce better results [53]. U-Net has more spatial information in the earlier stages of the network, and this spatial information provides a rich context to the following stages via skip connections. However, with this process, poor representations from the previous stages also come with good ones. To overcome the problem, the attention mechanism is implemented to skip connections to suppress activations from unrelated areas of the image. In the study, the attention mechanism proposed by Oktay et al. [26] was implemented in the Residual U-Net model (Figure 3).

#### 2.2.4. Inception Backboned U-Net

Szegedy et al. [21] proposed the first version of Inception in 2015 as GoogleNet. Their primary aim was to improve the computing resource usage of the model. They increased the depth of the model, along with the width, and kept the computational cost constant. They assumed that each unit of the earlier layers corresponds to some relevant region of the input image, and these are grouped into filter banks. Thus, they proposed many clusters concentrated in a specific region, and then covered them with 1 × 1 convolutions in the next layer. However, it would be expected that there will be spatially spread out clusters that can be covered by convolutions over large patches and a decrease in the number of patches over the larger regions [21]. Thus, the Inception architecture is restricted to 1 × 1, 3 × 3, and 5 × 5 filter sizes. Additionally, since pooling operations have been generally used in convolution networks, they added a pooling path in each Inception step. In the study, the encoder of the U-Net architecture was replaced with the Inception-v1 architecture, and convolution blocks in bottleneck and decoder paths were replaced with residual convolution blocks to generate the model.

#### 2.2.5. Inception-ResNet Backboned U-Net

One of the examples of the widely used deep CNNs is the Inception architecture [21], which achieves successful results at a low computational cost. Lately, the residual connections [20] have provided accomplished results in computer vision tasks. This raised a question for Szegedy et al. [54] of whether there would be a benefit in combining Inception networks with residual connections or not. They studied this idea, and proposed the Inception-ResNet architecture [54].

They thought that, since Inception networks are very deep, it would be reasonable to replace the filter concatenations with residual connections. For the residual version of Inception, they used cheaper blocks than the original. Each Inception block is followed by a 1 × 1 convolution without the activation as a filter expansion layer to scale up the dimensionality, prior to residual addition. In the study, the encoder of the U-Net architecture was replaced with the Inception-ResNet-v2 architecture, and convolution blocks in bottleneck and decoder paths were replaced with residual convolution blocks to generate this model.

#### 2.2.6. Xception Backboned U-Net

François Chollet presented an Inception interpretation as a step between conventional convolutions and depthwise separable convolutions, called “Xception” [55]. It is possible to think of the depthwise separable convolution as an Inception module with a lot of towers [55]. The idea prompted them to suggest a deep CNN modeled after Inception, by using separable convolutions in place of the Inception module [55]. This extreme version of the Inception module firstly maps the cross-channel correlations by using a 1 × 1 convolution; after that, it maps every output channel’s spatial correlations separately. In the study, the encoder of the U-Net architecture was replaced with the Xception architecture, and convolution blocks in bottleneck and decoder paths were replaced with residual convolution blocks to generate the model.

#### 2.2.7. MobileNet Backboned U-Net

Howard et al. presented mobile and efficient models called “MobileNets” in 2017 [56]. MobileNets are based on streamlined architectures that use depthwise separable convolutions to make the model lightweight. Their structure is built on depthwise separable convolutions for less expensive processing, except for the first layer, which is full convolution [56]. In the models, Batch Normalization and ReLU are placed after each convolution layer. Downsampling is performed with the strided convolution in depthwise convolutions, and in the first convolution layer. To lower the spatial resolution prior to the final output layer, an average pooling is utilized at the end of the model. In the study, the encoder of the U-Net architecture was replaced with the MobileNet-v1 architecture, and convolution blocks in bottleneck and decoder paths were replaced with residual convolution blocks to generate this model.

#### 2.2.8. Trans U-Net

For various image segmentation tasks, u-shaped CNNs achieved significant success. However, due to the fundamental locality of convolutions, these architectures are demonstrating some limitations [47]. Nonetheless, Transformers have been developed as alternative approaches with self-attention mechanisms. However, due to insufficient low-level details, they can result in limited localization abilities. Chen et al. [47] proposed Trans U-Net, which utilizes both U-Net and Transformers, as an alternative for computer vision tasks. On one side, the Transformer encodes tokenized image patches from the CNN feature map as input sequences. On the other side, the decoder upsamples these encoded features, which are combined with high-resolution feature maps to achieve accurate and precise localization [47].

Chen et al. [47] conducted a study on different model sizes and investigated two configurations, as “Base” and “Large” models. These two are separated from each other by size differences due to hyperparameter differences. However, in our study, a smaller variant is used, due to the computational resource limitations. We scaled the model according to the ratios of hyperparameters between the base and the large models, and used a variant named as “Mini”, referred in Table 1.

#### 2.2.9. Swin U-Net

Cao et al. [57] proposed Swin U-Net in 2021, a U-Net-like Transformer approach. In Swin U-Net, tokenized image patches are fed into a u-shaped, Transformer-based, encoder–decoder architecture that uses skip connections. They used Swin Transformers with shifted windows (swin) as an encoder for feature extraction. Motivated by the Swin Transformers [46], they proposed Swin U-Net to enhance the power of Transformers for image segmentation. Swin U-Net is the first u-shaped, Transformer-based architecture that consists of an encoder, a decoder, and a bottleneck, which are built on the Swin Transformer block, with skip connections [57].

#### 2.2.10. Residual-Inception U-Net (RIU-Net)

In this study, we proposed a residually connected, Inception-based, u-shaped encoder–decoder architecture with skip connections named as Residual-Inception U-Net (RIU-Net). The model includes an encoder path, a bottleneck, and a decoder path. For the encoder path of this architecture, a modified Inception-ResNet-v2 network [54] was used.

Throughout the network, as differs from the Inception-ResNet-v2, “Layer Normalization” [58] was used as a regularization technique instead of Batch Normalization, and the “GELU” activation function [59] was used instead of ReLU. The ReLU activation function is widely used in CNNs due to its efficiency and simplicity. GELU, which is a smoother variant of ReLU, is used in the most advanced Transformer approaches, and most recently in ViT [48]. As a result of the experiments, it was seen that the use of GELU instead of ReLU, which is widely used in computer vision studies, positively affects the accuracy. Batch Normalization is an important component of CNNs, since it improves convergence and reduces overfitting. However, Batch Normalization also has many complexities that can reduce the performance of the model [48]. There have been several attempts to develop an alternative, but Batch Normalization remained as the most commonly used one for many computer vision tasks. On the contrary, Layer Normalization, which is a simpler variant, has been used in Transformers, and it resulted in a good performance across many different applications [48]. In our study, the replacement of Batch Normalization with Layer Normalization resulted in a significant improvement in the accuracy of the proposed model. All convolution blocks used in the architecture include a convolution layer, Layer Normalization, and GELU activation, respectively, unless otherwise stated. These modifications are inspired by Liu et al. [48], where they “modernize” the CNNs toward the design of ViT.

**Figure 4:**
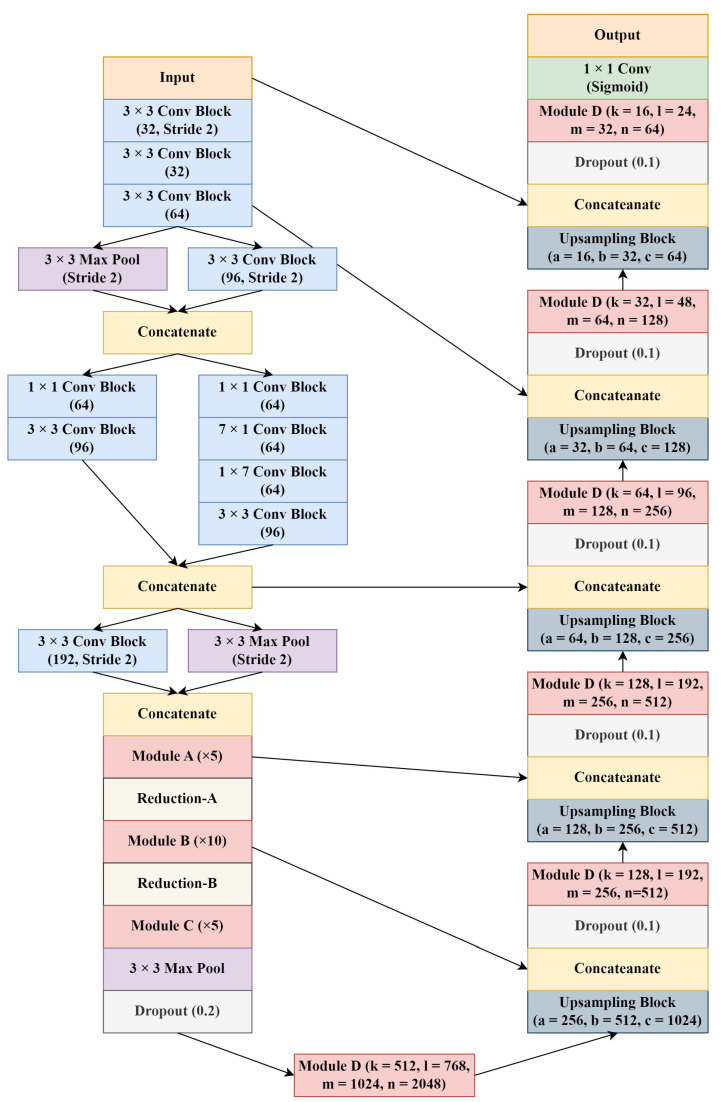
Overall architecture of the RIU-Net.

The overall design of the proposed approach is given in Figure 4. For the first part of the encoder, STEM design from the Inception-ResNet-v2 was used, with slight modifications. All “Valid” paddings used in the STEM were replaced with “Same” paddings. For the rest of the encoder, the modules used in Inception-ResNet-v2 were modified and implemented into the architecture. The Inception approach has achieved satisfactory performance at a low computational cost. In addition, combining them with residual connections significantly accelerated the training of Inception-based models. Since the Inception networks tend to be deep, it is beneficial to use residual connections instead of conventional convolutional connections. The use of residual connections helps to solve the vanishing gradient problem that exists in deep networks [27]. It allows Inception to acquire all the benefits of the residual connection while maintaining the computational efficiency [54].

**Figure 5:**
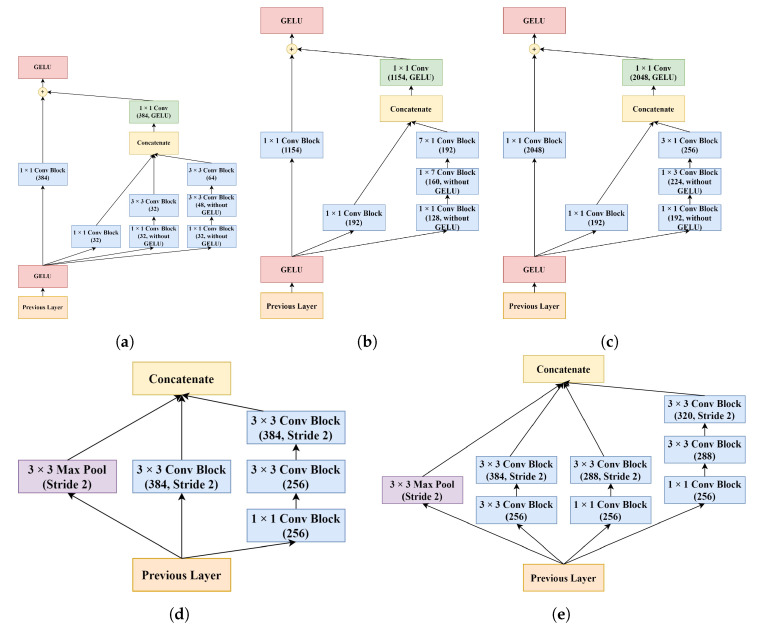
The flow diagram of the modules used in the encoder path of the RIU-Net: (**a**) Module A, (**b**) Module B, (**c**) Module C, (**d**) Reduction A, and (**e**) Reduction B.

The main modules A, B, and C were generated by modifying the Inception-ResNet-v2 modules. These modules were implemented in the encoder after the STEM. In these main modules, 1 × 1 convolution blocks were added and applied to the data coming from the previous layers different from the original ones. Afterward, the outputs of these shortcut blocks and the outputs of the main Inception blocks were merged to achieve the residual connection. In addition, the use of asymmetric convolutions in the STEM and the main modules resulted in significant computational cost savings. Reduction modules A and B remained similar to the original ones, but with all “Valid” paddings being replaced with “Same” paddings. The designs of all modules used in the encoder path are shown in Figure 5. Lastly, at the end of the encoder path, the average pooling layer in the Inception-ResNet-v2 has been replaced with a max pooling layer.

**Figure 6:**
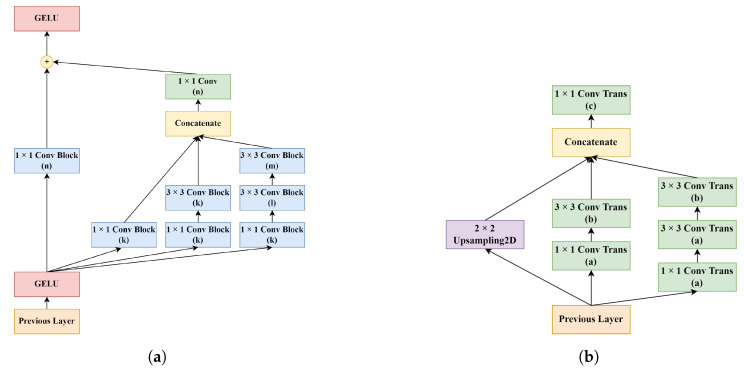
The flow diagram of the modules used in the bottleneck and decoder paths of the RIU-Net: (**a**) Module D, and (**b**) Upsampling module.

In the bottleneck and the decoder paths, residual Inception module D design was used with various numbers of filters. Additionally, instead of directly using a transposed convolutional layer, as was practiced in most CNN approaches, an Inception-based upsampling module was created and used in the decoder path. The direct use of max pooling and transposed convolutions to upsample the feature maps, such as in standard U-Net, may lead to accuracy reduction and feature loss [60]. Thus, upsampling modules have been preferred for overcoming these problems. The upsampling module was inspired by the paper from Zhang et al. [60] and implemented into the study with slight modifications. Lastly, between the encoder and the decoder paths, skip connections were used to avoid spatial information loss by transferring low-level feature maps from the encoder to the decoder. With these connections established between the relevant layers, low-level and high-level details were combined. The designs of Module D and the upsampling module are shown in Figure 6.

## 3. Experiments

Prepared training sets were used to train the generated models. Every experiment within this study was run on the cloud via the Google Colaboratory platform using an NVIDIA Tesla P100 GPU, and all the experiments, model developments, and implementations were performed using the Tensorflow framework. As mentioned previously, for all datasets, 70% of the data were used for training, 15% for validation during training, and the remaining 15% for testing. During the training process, as an optimizer, Adam [61] was used with a 1 × 10^−4^ initial learning rate. To reduce the learning rate during training, “ReduceLROnPlateau” callback was applied. Validation loss values were monitored by this callback, and the learning rate was decreased by a factor of 0.1 when the value did not improve for five consecutive epochs. The models were trained using a batch size of 2, and two loss functions were used together to calculate loss values. These loss functions are the Binary Cross-entropy Loss and the Jaccard Loss. After a series of experiments, including different loss functions and their combinations, it has been observed that a combination of these two functions provided the best results, according to the evaluations made. Binary Cross-entropy is a function to calculate the loss used on binary segmentation and classification tasks, and it is frequently used to measure how well the predicted class probabilities match the correct classes (Equation (1)) [62]. Jaccard Loss, IoU Loss, or Jaccard Index, is a ratio between the intersection of the predicted and ground truth and the union of these two (Equation (2)) [62]. Combining two loss functions allows for diversity in the loss while taking the advantage of the stability of Binary Cross-entropy. To determine the number of epochs for the models to train, a callback called “EarlyStopping” was used with a maximum of 50 epochs. Validation loss values were monitored and if the loss value did not improve for 10 consecutive epochs, the training was stopped. Another callback, the “ModelCheckpoint”, was also used during training. With this function, the model was saved each time the loss value improved during training, and then the best model was selected for testing and evaluation. The epoch of the best models and the number of trainable parameters for all models are presented in Table 2.

(1) $$L\left(y,\hat{y}\right)=\frac{1}{N}+\sum_{i=0}^{N}\left(y*\log\left(\hat{y_{i}}\right)+\left(1-y\right)*\log\left(1-\hat{y_{i}}\right)\right)$$

(2) $$Jaccard\left(A,B\right)=1-\frac{|A\cap B|}{|A\cup B|}=1-\frac{|A\cap B|}{|A|+|B|-|A\cap B|}$$

After the training, the best models were selected, and tests and evaluations were made using the prepared test sets. Metrics for evaluation were calculated, and the images were segmented using a 0.5 threshold applied to the predicted class probabilities. For the evaluation of the models; Recall (Equation 3), Precision (Equation 3), IoU (Equation 4), and F1 Score (Equation 5) evaluation metrics were used. F1 score, which is the harmonic mean of both Precision and Recall, is an evaluation metric used to assess a model’s overall performance [63]. IoU is a metric that gives the ratio of the intersection of the predicted area and the labeled area to the sum of these two areas [62]. These two metrics were taken as the main evaluation metrics to evaluate the models, as they are more suitable for image segmentation tasks. Along with the mentioned metrics, test loss and test accuracy were also calculated.

(3) $$\mathrm{Precision}=\frac{TP}{\left(TP+FP\right)}\;\mathrm{Recall}=\frac{TP}{\left(TP+FN\right)}$$

(4) $$IoU=\frac{|A\cap B|}{|A\cup B|}=\frac{TP}{\left(TP+FP+FN\right)}$$

(5) $$F1\;\mathrm{Score}=2*\frac{\mathrm{Precision}*\mathrm{Recall}}{\mathrm{Precision}+\mathrm{Recall}}$$

## 4. Results and Discussion

**Figure 7:**
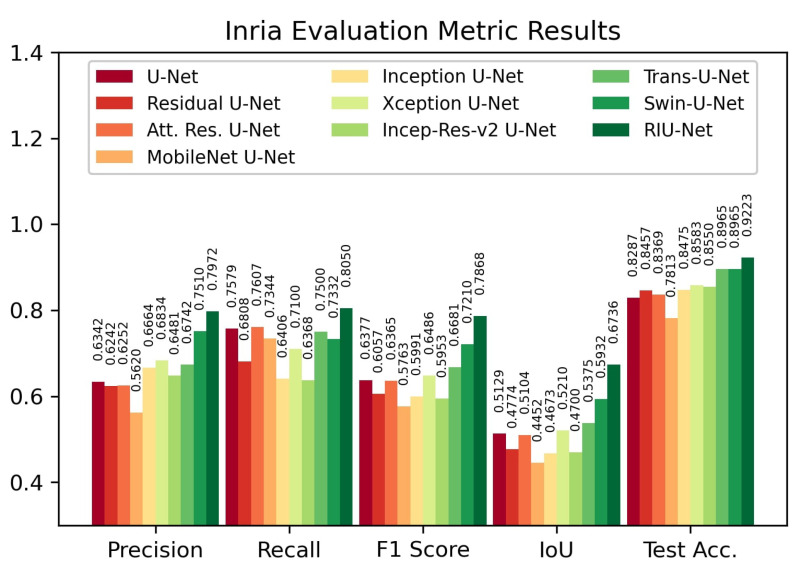
Evaluation metric results on Inria test set.

Evaluation results on the Inria test set are shown in Figure 7. According to the results, our proposed RIU-Net approach has the highest IoU score, F1 score, and test accuracy, with 0.6736, 0.7868, and 92.23%, respectively. The Transformer models Swin U-Net and Trans U-Net follow this approach. Swin U-Net has a 0.5932 IoU score, a 0.7210 F1 score, and 89.65% test accuracy. Trans U-Net follows, with 0.5375 IoU score, 0.6681 F1 score, and 89.65% test accuracy. For all three metrics, the MobileNet backboned U-Net has the lowest values, with 0.4452 IoU score, 0.5763 F1 score, and 78.13% test accuracy.

**Figure 8:**
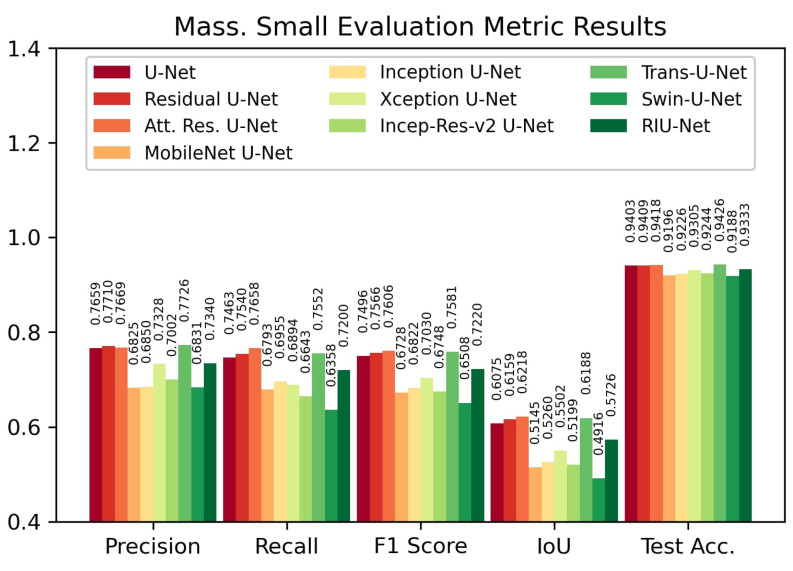
Evaluation metric results on Massachusetts Small test set.

Evaluation results on Massachusetts Small test set are shown in Figure 8. On this test set, the Attention Residual U-Net model provided the highest IoU score with 0.6218 and the highest F1 score with 0.7606. On the contrary, the Trans U-Net model provided the highest test accuracy with 94.26%. For the IoU score and F1 score metrics, Trans U-Net follows Attention Residual U-Net with 0.6188 and 0.7581, respectively. Residual U-Net follows Attention Residual U-Net with a 0.7566 F1 score and 0.6159 IoU score.

**Figure 9:**
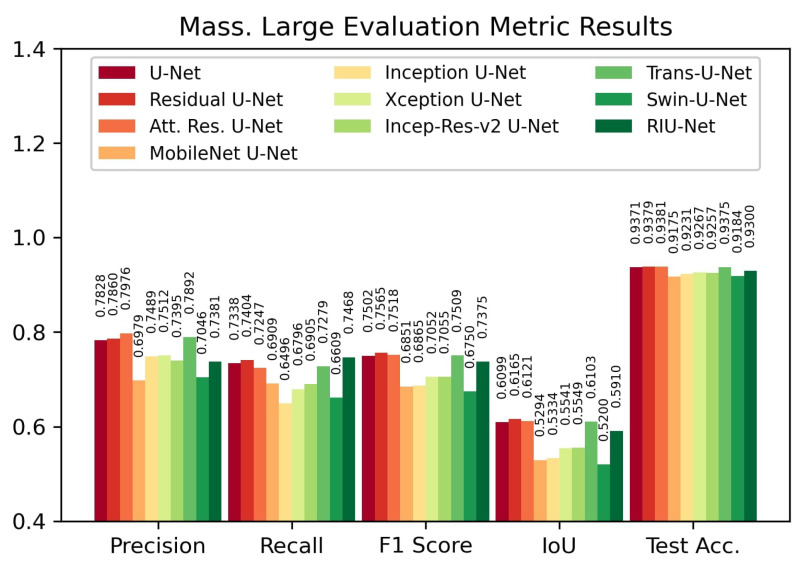
Evaluation metric results on Massachusetts Large test set.

According to the results on Massachusetts Large test set, shown in Figure 9, the Residual U-Net model provided the highest IoU score with 0.6165 and F1 score with 0.7565. This is followed by Attention Residual U-Net with 0.6121 IoU score and 0.7518 F1 score, and Trans U-Net with 0.6103 IoU score and 0.7509 F1 score. On both Massachusetts test sets, Swin U-Net and MobileNet backboned U-Net models have the lowest evaluation metric values.

**Figure 10:**
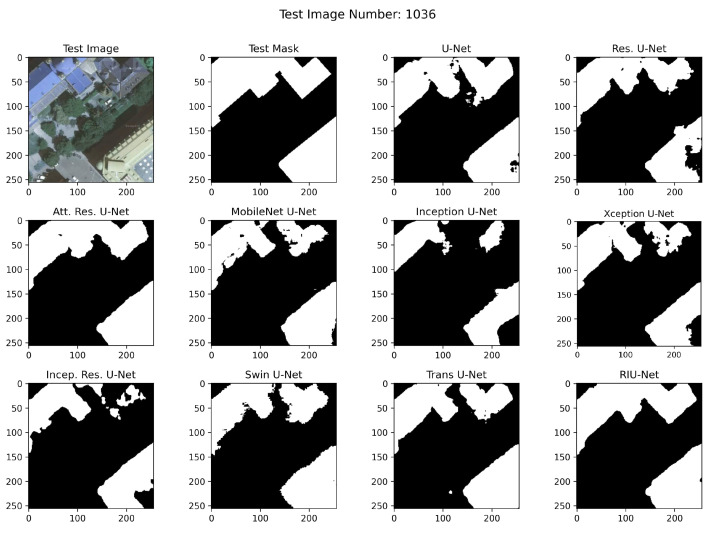
Inria test set image no. 1036 segmentation results.

**Figure 11:**
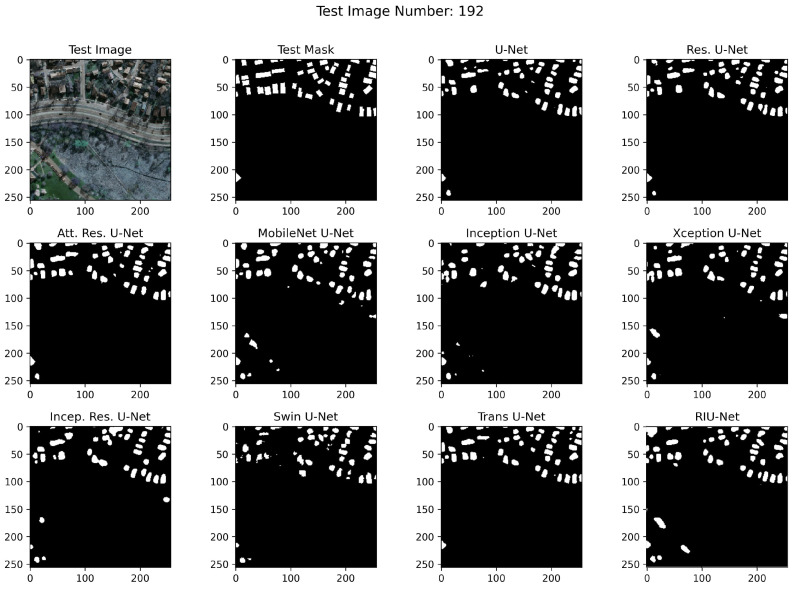
Massachusetts Small test set image no. 192 segmentation results.

**Figure 12:**
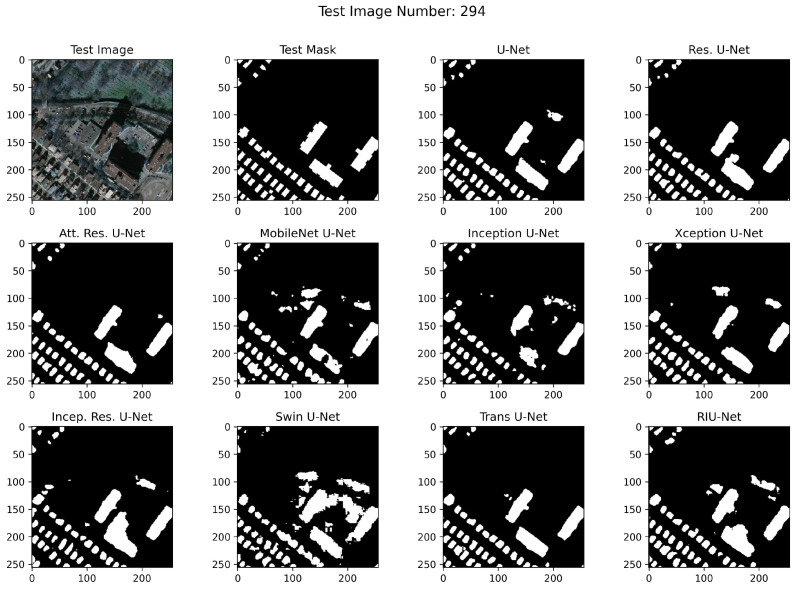
Massachusetts Large test set image no. 294 segmentation results.

RGB images from test sets, corresponding masks, and example segmented images are shown in Figure 10, Figure 11, and Figure 12, respectively. In the segmented images, black pixels represent the background and the white pixels represent the buildings. It has been observed that RIU-Net and Transformer approaches Swin U-Net and Trans U-Net have performed well according to the predictions on the Inria test set shown in Figure 10. Compared to other approaches used in the study, these models, especially the RIU-Net, are successful in segmenting the buildings with different colors, shapes, and textures in the related image no. 192. Other models have been found to have difficulty in distinguishing between dark building parts and trees, which are particularly similar in hue. According to the predictions on the Massachusetts test sets shown in Figure 11 and Figure 12, Trans U-Net, Attention Residual U-Net, and Residual U-Net have performed better than other approaches. Other models seem to classify non-buildings as buildings, or fail to classify some buildings. This problem is seen especially in areas where the shadow effect is high, or where buildings are in close color hue with the background.

On the Inria dataset, which includes buildings with different characteristics from different cities, the proposed RIU-Net approach has achieved significantly more successful results than other models in all metrics. On Massachusetts datasets containing images of buildings with similar characteristics from a single city (Boston, MA, USA), Residual U-Net, Attention Residual U-Net, and Trans U-Net models were found to give more successful results than the proposed approach. When the results obtained from the small and large datasets generated from the Massachusetts Buildings Dataset are compared with each other, it is seen that the RIU-Net indicates a significant increase in success compared to other models as the size of the dataset increases, and the difference between the models that give more positive results than themselves decrease. In addition, while the RIU-Net continues to learn when the 50-epoch limit is reached on all datasets, it has been observed that all other models stopped training with early stopping before this limit is reached. This demonstrates that longer-term training can improve the performance of the proposed approach even further.

On all the three test sets, it was observed that the MobileNet backboned U-Net model was among the least successful models, while the Trans U-Net model was among the top three successful models. It was seen that the Swin U-Net model gave the least successful results on Massachusetts datasets, whereas it was the second most successful model behind the proposed approach on the Inria dataset containing data with different characters. It is also seen that the proposed approach gives more effective results than the U-Net models using Inception, Xception, Inception ResNet, and MobileNet backbones in all three datasets.

It has also been seen that, in both the Inria dataset and the Massachusetts dataset, there are some inaccuracies. For example, buildings that are not in the images but are in the corresponding masks, buildings that are not fully visible in the images because they are covered with obstacles but contained in masks, and some missing labels.

In light of these results, it can be interpreted that the proposed RIU-Net approach provides more successful results than the models used in the study on data containing complex and different characteristic features, and on data containing more similar details, and that it starts to approach the performance of other models as the size of the dataset grows.

## 5. Conclusions

Over the past few years, automatic building segmentation and extraction from aerial images has become a significantly important subject due to the needs in application areas, such as city and regional planning, and change detection and disaster management, and the increase in usable data. Building segmentation was performed using generated datasets from the Massachusetts Buildings Dataset and the Inria Aerial Image Labeling Dataset within the context of the study. Ten CNN-based and Transformer-based models were generated, and their respective performance comparisons were subsequently made.

On the Inria dataset, which has buildings from various regions with different characteristics, the proposed RIU-Net model achieved the most successful results among all the models used. According to all evaluation metrics, RIU-Net performed significantly better than all other nine models, on this dataset. On the datasets generated using the Massachusetts Buildings Dataset, which includes buildings with similar characteristics and from the same region, Residual U-Net and Attention Residual U-Net, along with the Trans U-Net, one of the Transformer-based approaches, performed better. When the results obtained on the Small and Large Massachusetts datasets are compared, it is seen that the performance of the proposed RIU-Net model significantly increases compared to other models, as the size of the dataset grows. However, when the training process is examined, it has been observed that extending the training period may increase the performance of this proposed model.

When all the results are examined in general, it can be interpreted that the proposed RIU-Net approach is expressively successful on datasets containing complex buildings with different characteristics. On the other hand, on datasets containing buildings with mostly similar characteristics, it can be predicted that the proposed model will catch up with, and perhaps even exceed, the performance of other models used in the study when sufficient data and longer periods of training times are provided.

It is predicted that the proposed architecture can be used in classification and detection studies, in addition to segmentation studies, as in other CNN architectures used in literature. This approach can also be applied to other datasets for different image segmentation studies. In this study, as a result of the use of a u-shaped design with skip connections inspired by U-Net, which has achieved successful results in segmentation studies, together with Layer Normalization, GELU, Inception, residual connection, and asymmetric convolution approaches, a CNN model that can cope with Transformers, which have achieved successful results in recent years, has been proposed. This model approach has been achieved by modernizing existing and frequently used CNN approaches toward the ViT design.

In future studies, it is planned to test these models by conducting experiments with more data and longer training times. In addition to these, the creation of a more accurate dataset containing buildings from different regions and with various characteristics is also planned. It is also premeditated to train the models used in the study with large datasets containing buildings with different characteristics from different regions, and to share these trained models with researchers and potential users. In addition, to combine the best parts of both the CNN and Transformer approaches, it is planned to implement CNN–Transformer hybrid approaches, including our RIU-Net architecture, as the CNN part of the model. 

## Figures and Tables

**Table 1:** Hyperparameters of Large, Base, and Mini Trans U-Net variants.

Model Scale	Hidden Size (D)	No. of Layers	MLP Size	No. of Heads
Large	24	1024	4096	16
Base	12	768	3072	12
Mini	6	576	2304	9

**Table 2:** Number of trainable parameters and epochs of the best models.

Architectures	No. of Trainable Parameters	Epoch of the Best Model (Inria)	Epoch of the Best Model (Mass. Small)	Epoch of the Best Model (Mass. Large)
U-Net	31 M	27	29	25
Residual U-Net	32.4 M	10	19	21
Attention Residual U-Net	37.3 M	21	25	24
Inception backboned U-Net	373.1 M	6	16	26
Xception backboned U-Net	463.7 M	29	21	15
MobileNet backboned U-Net	102.8 M	12	16	17
Inception-ResNet backboned U-Net	164 M	10	25	17
Trans U-Net (Mini)	97.7 M	26	29	19
Swin U-Net	15 M	34	14	16
Residual-Inception U-Net (RIU-Net)	104.4 M	50	41	48

## Data Availability

Not applicable.
